# The Passing of 1907

**Published:** 1907-12-28

**Authors:** 


### Nursing Administration.

### THE PASSING OF 1907.

From a nursing point of view the event which has lent significance to 1907 is the King's choice of the founder of modern nursing for the first woman enrolled on his Order of Merit. In doing this he has not only honoured the lady whom all delight to honour in her beautiful old age, but he has honoured all the devoted women who have striven with single-hearted enthusiasm to tread in the road she marked out through the wilderness of remediable pain. Side by side with the great generals, the poets, thinkers, and statesmen of the world, we have in the estimate of merit attempted by King Edward, an estimate boldly differing from that of his predecessors or contemporaries, the woman who had the courage to devote herself, in the interests of humanity, to mechanical drudgery of the least inspired description, and to perceive that in the perfect performance of the meanest duties lay nobility of force to raise a nation. It is not merely that Miss Florence Nightingale organised the care of the wounded in the Crimean war. It is that she has had the breadth of view to foresee the part which women of education and refinement were to play in the development of hospital work, and to lay the basis of the new profession for women so soundly that countless others have been able to build securely ever since upon her foundation. The records of the year show a remarkable tale of women who have helped to rear the superstructure, women whose work stretches far back into the old days of pioneer training, and who in 1907 laid down their duties in happy consciousness of duty faithfully performed.

Seldom has a year brought within its narrow compass the retirement of so many whose names are household words in the history of nursing. In Scotland the Edinburgh Royal Infirmary has lost Miss Spencer, and the Glasgow Royal Infirmary Mrs. Strong, and the loss in both cases is irreparable, although the work done by each is of that undying character which is reflected in the enthusiasm of hundreds of well trained nurses, many of whom are themselves at the present time heads of training schools. Miss Calvert has retired from the Manchester Royal Infirmary, Miss Hunt from the Darlington Hospital, and Miss Chambers from the Ancoats Hospital, Manchester, after long periods of self-forgetting labour, during which their interests have become wholly identified with the hospitals they served. Three pioneer figures in Metropolitan Poor-law work have entered upon well-earned repose —Miss Malim, the founder of the Kensington Infirmary Training School; Miss Armit, whose labours at Southwark Infirmary are well known; and Miss Ramsden, from Marylebone Infirmary. It is not very creditable to our Poor-law system to reflect that two at least of these able and devoted superintendents were compelled to curtail their term of service owing to injury to health induced by the excessive pressure of work. Should Poor-law establishments ever fall under the management of able administrators it will be instantly recognised that there is small economy in wasting the brains and overtaxing the physique of their most valuable officers in order to save the cost of extra accommodation or the salaries of a few additional nurses. At the Fever Hospital Miss Ingall has completed an unusually long and creditable career, and Miss Smedley, though her term at St. George's Hospital has not been long, has left many sincere regrets behind. Among those who have become identified with the interests of nurses the name of Miss McAlpin, hon. superintendent of the Glasgow Home for Nurses, must not be forgotten. We may be permitted to wish a full measure of peace and prosperity to these prominent workers in the nursing world in their new-found leisure. We cannot forget that it is to the matrons who have retired from active work that the profession owes some of the highest voluntary work undertaken in its interests.

We record with deep regret the names of some who have passed away this year in the midst of their labours. Miss Guthrie Wright, the founder of the Scottish Branch of the Jubilee Institute, has left her residence to be used as a Home of Rest for Nurses, and it is hoped shortly to raise an endowment to preserve this as a permanent memorial of her labours. The name of Miss Bromhead, whose work for nurses in Lincoln has extended over so long a period, is to be associated with a much needed home for nurses in that city which is being built as a memorial to her. Miss Bulteel, Matron of the Taunton and Somerset Hospital, and Miss Glover, Matron of the Royal Albert Hospital, have both died almost in harness, their loss being felt as a personal grief by those privileged to work under their superintendence.

There is no need to feel anxious about the future of nursing so long as the profession continues to attract the services of women such as we have enumerated, women who can boast as their head her who in the judgment of the King stands foremost among her sex in the Empire. If sometimes those who are working most zealously in the interests of nurses grow discouraged at the continuance of abuses, and at the want of coherence among those who are working with a common aim, they would do well to reflect that the future of the nursing profession depends primarily on the character of those who compose it, and very little, if at all, on the carrying of resolutions. It is easy to point to a hundred instances of bodies organised with admirable skill and forethought, which yet are decaying inwardly and exhibit every sign of defective vitality. On the other hand nursing, with no governing body, without a voice which can make itself heard, with no penal code, and no commonly accepted standard of training, has gathered under its wing some of the highest material available in the interests of humanity and is doing work which has never been equalled for value in the history of our race. Organisation will come when the time is ripe. Meantime we rejoice that our founder is still among us, and we claim participation in the honour she has received.

